# God is in His Heaven, All’s Right With the World: Psychological Well-being and Belief in Divine Control During the Third COVID-19 Lockdown Among Anglican Clergy and Laity in England

**DOI:** 10.1177/00916471221149027

**Published:** 2023-01-12

**Authors:** Andrew Village, Leslie J. Francis

**Affiliations:** York St John University, UK; University of Warwick, UK; Bishop Grosseteste University, UK

**Keywords:** psychology of religion, divine control, pandemic, positive affect, Anglican, negative affect

## Abstract

Drawing on data provided by 1,841 lay or ordained members of the Anglican Church residing in England during the first half of 2021, this study explores the connection between self-perceived change in psychological well-being during the pandemic and belief in divine control over the pandemic. Change in psychological well-being was assessed by The Index of Balanced Affect Change (TIBACh) that distinguishes between positive affect and negative affect, and divine control was assessed by the God in Control of the Pandemic Scale (GiCoPS). After controlling for personal factors (age and sex), psychological factors (psychological type and emotional volatility), contextual factors (education level and ordination status), and ecclesial factors (conservative doctrine and charismatic influence), the data demonstrated a positive association between belief in divine control and change in positive affect, but no association between belief in divine control and change in negative affect.

## Introduction

Robert Browning’s aphorism ‘God is in his heaven, all’s well with the world’ raises an important question for empirical theology and for the empirical psychology of religion in the context of exploring the impact of religious beliefs and psychological well-being of clergy and lay church members during the pandemic. The extent and impact of the rapidly developing COVID-19 pandemic at the beginning of 2020 reached every aspect of human daily experience, and churches and religious institutions were no exception. In England, the government imposed a lockdown on the nation on 23 March 2020. Shops and industries were shut down. Schools and universities were closed. All but key workers were instructed wherever possible to work from home. Setting an example for the nation, the Church of England locked its churches (except for key services like the operation of foodbanks). Clergy were not allowed to enter their churches to celebrate communion. Lay people were not allowed to enter their churches for personal prayer.

With Robert Browning’s aphorism in mind, the aim of this study is to examine the perceived impact of the pandemic on well-being among Anglican clergy and laity in England and the connection between change in well-being and belief in divine control during the pandemic. The Anglican tradition inherited the notion of divine providence, which was widespread in early modern England (Walsham, 1999). Central to this notion was the belief that God acts in history in a general way, but also in specific events. The *Book of Common Prayer* reflects this idea in some of the special petitionary prayers, which includes one to be used in ‘The time of any Plague of Sickness’:

O ALMIGHTY God, who in thy wrath didst send a plague upon thine own people in the wilderness, for their obstinate rebellion against Moses and Aaron; and also, in the time of king David, didst slay with the plague of pestilence threescore and ten thousand, and yet remembering thy mercy didst save the rest: Have pity upon us miserable sinners, who now are visited with great sickness and mortality; that like as thou didst then accept of an atonement, and didst command the destroying Angel to cease from punishing, so it may now please thee to withdraw from us this plague and grievous sickness; through Jesus Christ our Lord. **Amen.**

The practice of national fasting in times of plague continued into the 19th century, demonstrating “the wide popular acceptance of belief in providence and an interventionist God” (Janet, 1982, p. 297). Although belief in the idea of God acting miraculously in the word was challenged by the liberal wing of the Church of England (Robinson, 1963), it was still a hotly debated issue when David Jenkins, a vocal critic of the idea of divine intervention, was ordained Bishop of Durham in 1984 (Dyson, 1985).

The context for this new enquiry is shaped in four steps. Step 1 locates the present enquiry against the background of the *Coronavirus*, *Church & You Survey* conducted during the first national lockdown that provided a firm foundation for research among Anglicans during the pandemic. Step 2 examines the balanced affect model of psychological well-being adopted by the *Coronavirus, Church & You Survey* and introduces the development of The Index of Balanced Affect Change (TIBACh) that is employed in this study. Step 3 turns attention to the *COVID-19 & Church-21 Survey* conducted during the third national lockdown and introduces the new set of items included in this survey and on which the paper builds to develop a new measure, the God in Control of the Pandemic Scale (GiCoPS). Step 4 explores why the examination of the relationship between scores recorded on TIBACh and GiCoPS were contextualized within specific control variables.

### Introducing the *Coronavirus, Church & You Survey*

In response to such rapid changes impacting the lives and experiences of clergy and lay people, the *Coronavirus, Church & You Survey* was developed in partnership between York St John University and the *Church Times*, the main Anglican newspaper in England. It was promoted both by the *Church Times* and by a number of Anglican dioceses in England. This survey, launched during the first national lockdown, was open online between 8 May and 23 July 2020 and attracted responses from over 7,000 participants, of whom 5,347 identified as Anglicans living in England. Data from the *Coronavirus, Church & You Survey* have been used to examine a range of focused research questions concerning the responses of clergy and lay people to the pandemic during that initial lockdown. For example, one set of studies focused attention on the impact of the pandemic exacerbating the fragility of rural Anglican churches through the eyes of clergy (Francis et al., 2020) and lay people (Francis, Village, & Lawson, 2021), and explored the consequences for how rural lay people viewed the leadership of the Church of England during the pandemic (McKenna, 2021). A second set of studies compared the responses of different groups of clergy, comparing the perspectives of stipendiary and retired clergy (Francis & Village, 2021e) and comparing the perspectives of Anglo-Catholic and Anglican Evangelical clergy (Francis & Village, 2022b). A third set of studies compared the responses of different groups of lay people, focusing on age difference (Francis & Village, 2021c) and sex differences (Francis & Village, 2022a). A fourth set of studies examined the responses to the migration to online worship (Francis & Village, 2021d; McKenna, 2022) and the impact of closing churches for worship (Village & Francis, 2021a). Other studies examined spiritual awakening during the pandemic among clergy (Francis, Village, & Lewis, 2021) and among lay people (Francis, Village, & Lewis, 2022) and the role of psychological type preferences in shaping responses to the pandemic (Francis & Village, 2021b; Village & Francis, 2021c).

### Assessing change in psychological well-being

One key question in the *Coronavirus, Church & You Survey* assessed the impact of the pandemic on the psychological well-being of clergy and lay people. This question raised two conceptual and operational issues. The first issue concerned a clear understanding of the definition and measurement of psychological well-being. The approach adopted by the *Coronavirus, Church & You Survey* drew on the classic model of balanced affect as proposed by Bradburn (1969). This model maintains that good psychological well-being is a function of the balance between positive affect and negative affect. The model recognizes that positive affect and negative affect are not opposite ends of a simple continuum but operate as two partially independent continua. According to this model, it is possible for an individual to record high scores of positive affect and at the same time record high levels of negative affect. Most recently this model has been employed to underpin the Francis Burnout Inventory (Francis et al., 2005) where for clergy negative affect is operationalised as emotional exhaustion in ministry, and positive affect is operationalised as satisfaction in ministry. A series of studies among different groups of clergy has demonstrated that, for some, high emotional exhaustion can be accompanied by high satisfaction in ministry, and that high satisfaction in ministry can mitigate the deleterious effects of high emotional exhaustion (Francis, Crea, & Laycock, 2017, 2021; Francis et al., 2011, 2019; Francis, Laycock, & Brewster, 2017; Francis, Laycock, & Crea, 2017; Village et al., 2018).

The second issue concerned establishing a satisfactory method for assessing change in psychological well-being caused by the pandemic. Scientifically, the most effective way of doing this would have been by recording levels of positive affect and levels of negative affect among the same individuals both before the pandemic and after the pandemic had taken hold. Clearly, this was not a viable option. It is for this reason that the *Coronavirus, Church & You Survey* proposed a new measure that we styled The Index of Balanced Affect Change (TIBACh). In the foundation paper for this instrument, Francis and Village (2021a) describe the process whereby the TIBACh was developed.

The *Coronavirus, Church & You Survey* contained a pool of 20 items introduced by the following rubric: “How would you rate the effect of the lockdown on you so far?, (Please click one button EACH row to indicate a positive (+) or negative (-) effect. The middle button (0) indicates no effect of the lockdown).” Items were presented on a three-point bipolar scale with radio buttons between them to indicate if that aspect of well-being had declined, increased, or remained unchanged during the lockdown. In the foundation paper for the TIBACh (Francis & Village, 2021a), exploratory factor analysis (principal components extraction and varimax rotation) indicated two factors that represented positive affect (excitement, thankfulness, happiness, hopefulness, and trust) and negative affect (exhaustion, anxiety, stress, fatigue, and frustration). The resulting two 5-item scales generate acceptable internal consistency reliability, with Cronbach alphas of .70 for positive affect and .83 for negative affect. Positive affect items were coded such that a high score indicated an increase in positive aspects of well-being during the lockdown; negative affect items were coded such that a high score indicated an increase in negative aspects of well-being during the lockdown. These two measures of positive affect and negative affect were negatively correlated (*r* = –.53, *p* < .001), but this was not a perfect correlation, which suggests that the two types of affect were not operating as a mirror image of each other. This raises the possibility that they may be related to slightly different sets of predictor variables.

In the foundation paper for the TIBACh, Francis and Village (2021a) established the construct validity of this balanced affect model against an independent measure of coping during the pandemic. Participants were invited to rate their personal response to the coronavirus crisis on a 5-point bipolar scale anchored at one end with “coped very poorly” (=1) and the other end with “coped very well” (=5). Bivariate correlations were in the expected directions: coping was positively correlated with positive affect (*r* = .46, *p* < .001) and negatively correlated with negative affect (*r* = –.49, *p* < .001). Crucially for the balanced affect model, there was a significant interaction effect of positive and negative affect on coping with the COVID-19 lockdown. The negative slope of coping against negative affect was steeper among those with below-average levels of positive affect and shallower among those with above-average levels of positive affect. This meant that, while there was little difference in average coping levels among those with low negative affect whatever their level of positive affect, for those with high negative affect, coping was higher among those with higher positive affect.

### Assessing belief in divine control during the pandemic

When the third national lockdown took hold during January 2021, the partnership between York St John University and the *Church Times* launched a second survey, the *Covid-19 & Church-21 Survey* that was open online between 22 January and 23 July 2021. This second survey re-ran some of the measures employed in the earlier survey, including the TIBACh, for comparative purposes. The second survey also wanted to explore issues that had not been raised in the earlier survey, and foremost among these issues was the theological question concerning the prevalence of belief in divine control during the pandemic. In particular, this survey was designed to explore the extent to which belief in divine control during the pandemic impacted psychological well-being as operationalised by the TIBACh.

It is for this reason that the *Covid-19 & Church-21 Survey* included a section headed by the following rubric: “Some people wonder about the role of God in the pandemic. What do you think?” This section then presented a set of nine statements, largely culled from the church press since the pandemic began:

- God has always been in control during the pandemic.
- God sent the pandemic to test our faith.
- God will save us from the pandemic through science.
- The pandemic is a solely “natural” event without any relation to God.
- God could stop the pandemic at any point whatever we do.
- The pandemic is punishment from God.
- God’s power to save us from the pandemic depends on human co-operation.
- Science will save us from the pandemic without God’s help.
- The pandemic is the result of human sin.

### Introducing the control variables

Previous research has demonstrated that individual differences both in religiosity and in well-being are related to personal factors, psychological factors, ecclesial factors, and contextual factors. In terms of personal factors, sex differences have long been established in the literature (Argyle & Beit-Hallahmi, 1975; Beit-Hallahmi & Argyle, 1997; Schnabel, 2015). More recent work has drawn attention to the importance of age, both as reflecting birth cohort effects and as reflecting the effects of aging per se (Village, 2018).

In terms of psychological factors, attention has been drawn to the importance of personality (Francis, 2005), drawing in particular on the three major dimensions (Eysenck & Eysenck, 1991), the big five factors (Costa & McCrae, 1992), and psychological type theory (Keirsey & Bates, 1978; Myers & McCaulley, 1985). With the development of the Francis Psychological Type Scales, psychological type theory has emerged more prominently within the fields of the empirical psychology of religion and empirical theology (Lewis, 2012, 2015, 2021a, 2021b; Village, 2011).

In terms of ecclesial factors, the differences between Anglo-Catholic, Broad Church, and Evangelical wings of the Church of England have long been understood and widely researched (Randall, 2005). A further nuance is provided by taking into account the effect of charismatics within the Church of England (Randall, 2005). In the Church of England, Anglo-Catholics have traditionally been conservative in matters of liturgy and ritual, but more liberal in terms of doctrine and moral issues. Evangelicals and Charismatics tend to be the opposite in holding to more traditional views on doctrine and moral issues. Broad Church refers to those who do not identify with either of these wings of the Church, and whose views tend to fall somewhere between the two extremes (Village, 2012, 2018). In terms of contextual factors, both level of education and ordination status have been shown to be important predictors of individual differences in religious beliefs and attitudes (Village, 2018).

Drawing on data from the *Coronavirus, Church & You Survey*, Village and Francis (2021b) employed the TIBACh to test the personal, psychological, contextual, and ecclesial correlates of individual differences in psychological well-being among clergy and laity. Data from this analysis, drawing on 4,449 participants, confirmed that personal factors were important (especially age, with older participants reporting a better outcome); psychological factors were important (especially emotional volatility and extraversion, with stable extraverts reporting a better outcome); ecclesial factors were important (especially church orientation, with Anglo-Catholics reporting a worse outcome and Anglican Evangelicals reporting a better outcome compared with Broad Church participants); and contextual factors were important. These findings confirm the importance of taking personal, psychological, contextual, and ecclesial factors into account as control variables in future research exploring the power of other predictor variables on individual differences in perceived change in well-being during the pandemic.

### Research aim

Against this background, this study was designed to address two primary issues. The first issue concerned developing a new measure to assess belief in divine control during the pandemic. The second issue concerned exploring the association between individual differences in belief in divine control during the pandemic and positive affect and negative affect as operationalised by the TIBACh. In light of what is already known about predictors of individual differences in positive affect and negative affect change, the second issue needs to be contextualised alongside relevant control variables, including personal factors, psychological factors, ecclesial factors, and contextual factors.

## Method

### Procedure

During the third lockdown in England, an online survey was promoted through the online and paper versions of the *Church Times*, the main newspaper of the Church of England, as well as directly through Church of England dioceses. The survey, named *Covid-19, & Church-21*, was delivered through the Qualtrics XM platform and was available from 22 January to 23 July 2021. It was designed to be used by various denominations. Within the total response from 5,853 participants, there were 1,841 clergy and lay people who identified as Anglican, lived in England, and had completed sufficient responses to be included in the following analyses.

### Participants

The 1,841 participants were comprised of 55% women and 45% men, and the majority (85%) were aged 50 or over (Table 1). Anglo-Catholics comprised 29% of the sample, Broad Church 51%, and 20% Evangelical. Over one-third (38%) were ordained.

**Table 1.** Sample Profile.

		%
Sex	Male	44.9
	Female	55.1
Age	20s	1.4
	30s	4.1
	40s	9.7
	50s	20.1
	60s	34.7
	70s	25.6
	80s+	4.5
Church tradition	Anglo-Catholic	29.1
	Broad Church	50.6
	Anglican Evangelical	20.4
Ordained status	Laity	62.5
	Clergy	37.5
Education	No formal qualifications	0.4
	School-level	4.6
	Certificate/diploma	15.8
	Bachelor degree	40.0
	Masters degree	29.5
	Doctorate	9.8

*N* = 1,841.

### Instruments

#### The role of God in the pandemic

The survey included a section of Likert-type items examining a range of attitudes toward the pandemic and its likely aftermath. One section of nine items was headed by the following rubric: “Some people wonder about the role of God in the pandemic. What do you think?,” which was followed by statements such as “God has always been in control during the pandemic,” “The pandemic is punishment from God,” and “Science will save us from the pandemic without God’s help.” Each had a 5-point response scale ranging from “strongly agree” to “strongly disagree.” The items were designed to give rise to the construction of a new measure, the GiCoPS.

#### TIBACh

The survey contained the two 5-item scales of TIBACh that measured changes in positive and negative affect during the pandemic (Francis & Village, 2021a; Village & Francis, 2022a). They were introduced with the statement “How would you rate how you are now compared with before the pandemic started?”. Respondents were asked to indicate on a 5-point response scale if positive affect (such as happiness), or negative affect (such as anxiety) had increased, stayed the same, or decreased. The scales had good internal reliability as measured by Cronbach’s alpha (positive affect = .78, negative affect = .82).

#### Personal variables

Personal variables were sex (0 = male, 1 = female) and age (by decade, 2 = 18–29 to 8 = 80+).

#### Psychological variables

Psychological type and emotional volatility were assessed using the revised version of the Francis Psychological Type and Emotional Temperament Scales (FPTETS) (Village & Francis, 2022b). This 50-item instrument comprises four sets of 10 forced-choice items related to each of the four components of psychological type theory (Extraversion-Introversion, Sensing-Intuition, Thinking-Feeling, and Judging-Perceiving), alongside 10 items related to emotional temperament (Calm-Volatile). The volatility scale has been shown to correlate strongly with neuroticism as measured by the Eysenck Personality Questionnaire Revised Shortened version (Village & Francis, 2022b). Scores for Extraversion (E), Sensing (S), Thinking (T), Judging (J), and Emotional volatility (V) were used as predictor variables. Internal consistency reliabilities (alpha; Cronbach, 1951) in this sample were E: .84, S: .79, T: .76, J: .82, and V: .84. The FPTETS and its predecessor, the Francis Psychological Type Scales have been shown to predict a wide range of religious expression, attitudes, and beliefs (see, Francis, 2005; Village, 2019 and references therein).

#### Contextual variables

Two aspects of individual context were assessed: educational attainment (highest level, ranging from 1 = no formal qualifications to 6 = doctoral level) and ordination status (lay = 0, ordained = 1).

#### Ecclesial variables

Church tradition for Anglicans was assessed using a 7-point bipolar scale labeled “Anglo-Catholic” at one end and “Evangelical” at the other. It is a good indication of differences in belief and practice in the Church of England (Randall, 2005; Village, 2012) and was used to identify Anglo-Catholic (scoring 1–2), Broad Church (3–5) and Evangelical (6–7). A similar scale was also used to assess identification with Charismaticism (seven-point semantic scale with 1 = not Charismatic and 7 = Charismatic). A third scale measured preference for liberal versus conservative doctrine (seven-point semantic scale with 1 = liberal and 7 = conservative).

### Analysis

All analyses employed procedures in SPSS 28 (IBM Corporation, 2021). Bivariate correlations were used to identify significant correlations between the measures of negative and positive psychological affect change and the 13 predictor variables, and between the predictor variables themselves. Hierarchical multiple linear regressions were used to identify independent effects, and to see if the effects of GiCoPS were stable when other independent predictors were added to the model. GiCoPS and personal variables were added in model 1, psychological variables in model 2, contextual variables in model 3, and theological variables in model 4.

## Results

The first step in data analysis concerned the development of a new measure to assess belief in divine control during the pandemic. Factor analysis of the groups of items regarding the role of God in the pandemic (principal components extraction and varimax rotation) identified four items that loaded on the same factor. These items related to how far God was in control during the pandemic, and how far science, rather than God, would save us from its effects (Table 2). These items were used to create the 4-item index GiCoPS, which had an acceptable internal consistency reliability as measured by Cronbach’s alpha (Cronbach, 1951). Scores were normally distributed (*M* = 13.6, *SD* = 3.4) and ranged from 4 to 20, with a high score indicating belief that God had a high level of control over the pandemic. A high score on this scale implied a stronger general belief in divine omnipotency, which was expressed in this context as the notion that God is able to control events such as a pandemic. This tended to be associated with a lower sense that science or humans could control events, suggesting that divine omnipotency was conceived as God acting independently of human agents. These items also demonstrate that more than two-thirds of the participants (69%) believed that God has always been in control during the pandemic, and that more than one-third of the participants (36%) believed that God could stop the pandemic at any point whatever we do. Alongside these beliefs in divine control, 44% of the participants took the view that the pandemic is a solely “natural” event without any relation to God, and 12% of the participants took the view that science will save us from the pandemic without God’s help. Clearly, there was diversity of belief among this group of 1,841 Anglican clergy and lay people living in England.

**Table 2.** Properties of the God in Control of the Pandemic Scale (GiCoPS).

	CITC	Agree (%)	Not certain (%)	Disagree (%)
Cronbach’s alpha = .67				
God has always been in control during the pandemic	.49	69	20	11
The pandemic is a solely ‘natural’ event without any relation to God*	.42	44	24	33
God could stop the pandemic at any point whatever we do	.46	36	21	43
Science will save us from the pandemic without Gods help*	.45	12	23	65

*N* = 1,841.

CITC: Corrected Item-Total Correlation.

* These items were reverse coded to form the GiCoPS.

The second step in data analysis concerned examination of the bivariate correlations among the core variables (GiCoPS and the measures of change in positive affect and negative affect proposed by TIBACh) and the control variables, including personal factors (age and sex), psychological factors (extraversion, sensing, thinking, judging, and emotional volatility), contextual factors (education and ordination), and ecclesial factors (conservative doctrine and charismatic influence). Three features of the correlation matrix presented in Table 3 deserve commentary. Given the number of correlations tested at the same time, attention will be drawn only to those who reach the 1% level of probability.

**Table 3.** Correlation Matrix of Dependent and Predictor Variables.

		14	13	12	11	10	9	8	7	6	5	4	3	2
1	Negative affect	–.01	–.03	.10***	.06**	.31***	.02	–.01	.00	–.01	–.22***	.02	–.02	–.50***
2	Positive affect	.12***	.07**	.05*	–.06*	–.27***	–.06*	–.05*	–.11***	.10***	.11***	–.05*	.12***	
3	God in control	.32***	.38***	.11***	.01	–.06*	–.05*	–.03	–.02	.09***	–.15***	–.06*		
4	Female	.04	–.15***	–.17***	–.10***	.15***	.01	–.19***	.08***	.01	–.02			
5	Age	–.14***	–.11***	–.14***	–.20***	–.17***	–.01	–.01	.10***	–.01				
6	Extraversion	.16***	.03	.07**	–.06*	–.13***	–.20***	–.17***	–.19***					
7	Sensing	–.15***	.14***	–.24***	–.25***	.06*	.43***	.12***						
8	Thinking	–.07**	.10***	–.11***	.11***	.05*	.27***							
9	Judging	–.11***	.07**	–.12***	–.01	.07**								
10	Emotional volatility	–.05*	–.02	–.08**	.00									
11	Education	–.04	–.06**	.22***										
12	Ordained	.15***	.05*											
13	Conservative doctrine	.27***												
14	Charismatic													

*N* *=* 1,841.

* *p* < .05; **p < .01; ***p < .001.

First, in terms of belief in divine control during the pandemic scores recorded on the GiCoPS are correlated positively with change in positive affect but independent of change in negative affect. This finding may be of theoretical significance as will be discussed later. However, it is also the case that scores recorded on the GiCoPS are correlated with a number of the control variables. In particular, higher scores on the GiCoPS are associated with influence by the Charismatic Movement, with espousing conservative doctrine, with ordination, and with extraversion, while lower scores on the GiCoPS are associated with age.

Second, in terms of change in psychological well-being, the two scale of the TIBACh behave in different ways. Influence by the Charistmatic Movement is associated with positive change in positive affect, but independent of negative affect. Similarly, espousal of conservative doctrine is associated with positive change in positive affect but independent of negative affect. Higher levels of negative affect change were found among clergy, those with higher levels of education, higher emotional volatility, and younger people. Higher levels of positive affect change were found among those with lower emotional volatility, intuitive types, extraverts, and older people.

Third, attachment to the Charismatic Movement was associated with younger people, extraverts, intuitive types, feeling types, perceiving types, and clergy. Holding conservative doctrine was associated with charismatic influence, being male, younger people, sensing types, thinking types, judging types, and lower education levels.

This complex pattern of interrelationships demonstrates the importance of testing whether the connection between belief in divine control during the pandemic and change in positive affect and negative affect remained significant after taking the control variables into account.

The data presented from the multiple regression models in Table 4 (on negative affect) and Table 5 (on positive affect) confirm that the core finding from the bivariate correlations remains unchanged, after taking into account personal factors (age and sex), psychological factors (extraversion, sensing, thinking, judging, and emotional volatility), contextual factors (education level and ordination status), and ecclesial factors (conservative doctrine and charismatic influence). Belief in the control of God during the pandemic had no influence on changes in negative affect (such as exhaustion, anxiety, stress, fatigue, and frustration) but was positively correlated with changes in positive affect (such as happiness, excitement, thankfulness, hopefulness, and confidence).

**Table 4.** Hierarchical Linear Regression of Negative Affect.

	Model			
	1	2	3	4
God in control	–.05*	–.03	–.04	–.02
Female	.02	–.03	–.01	–.02
Age	–.23***	–.18***	–.17***	–.17***
Extraversion		.02	.03	.03
Sensing		.01	.03	.03
Thinking		–.03	–.02	–.01
Judging		.01	.00	.00
Emotional volatility		.28***	.29***	.29***
Education			.02	.01
Ordained			.10***	.10***
Conservative doctrine				–.04
Charismatic				–.01

*N* *=* 1,841.

* *p* < .05; ***p < .001.

**Table 5.** Hierarchical Linear Regression of Positive Affect.

	Model			
	1	2	3	4
God in control	.13***	.11***	.11***	.07**
Female	–.04	.00	.00	.00
Age	.13***	.09***	.08**	.09***
Extraversion		.04	.03	.02
Sensing		–.10***	–.12***	–.11***
Thinking		–.02	–.02	–.02
Judging		.02	.03	.03
Emotional volatility		–.24***	–.24***	–.24***
Education			–.07**	–.07**
Ordained			.02	.01
Conservative doctrine				.04
Charismatic				.07**

*N* *=* 1,841.

**p < .01; ***p < .001.

## Discussion and Conclusion

The present survey was designed during the third lockdown in England, responding to the COVID-19 pandemic, and open from 22 January to 23 July 2021. Drawing on data provided to this survey by 1,841 lay or ordained members of the Anglican Church residing in England, the present analyses were designed to explore the connection between self-perceived change in psychological well-being during the pandemic and belief in divine control over the pandemic. To test this association, the analyses were focused by two clear research tasks. The first task was developing a new measure to assess belief in divine control during the pandemic, since no instrument had been previously designed to address this specific area. The second task was examining the association between individual differences in belief in divine control during the pandemic and self-perceived change in well-being as conceptualized and operationalised by TIBACh that distinguishes between change in positive affect and change in negative affect. Four main conclusions can be drawn from these data and analyses.

The first conclusion concerns the development of the new instrument: the GiCoPS. This 4-item measure, comprising two positively voiced items and two negatively voiced items, demonstrated satisfactory internal consistency reliability for such a short instrument (α = .67) with satisfactory variability in item discrimination, with endorsement of the positive items ranging from 36% to 69% and with rejection of the negative items ranging from 33% to 65%. These four items also display a high level of face validity. The scale can be commended for further use, possibly in forms tailored to specific crisis events.

The second conclusion concerns the co-variants of scores recorded on the GiCoPS. Higher belief in divine control during the pandemic was associated with influence by the Charismatic Movement, espousing conservative doctrine, ordination, extraversion, and being younger. While the two strongest correlations with belief in divine control during the pandemic were conservative doctrine and the Charismatic Movement, the regression model on positive affect demonstrated that the GiCoPS was providing an independent perspective additional to the effects of conservative doctrine and charismatic influence.

The third conclusion concerns the validity of the affect balanced model of change in psychological well-being. These data support the validity in two ways. First, the two scales of the TIBACh related to the control variables in different ways. Charismatic influence was associated with positive change in positive affect, but independent of negative affect. Espousal of conservative doctrine was associated with positive change in positive affect, but independent of negative affect. Higher levels of negative affect change were found among clergy, those with higher levels of education, those with higher emotional volatility, and younger people. Higher levels of positive affect change were found among those with lower emotional volatility, intuitive types, extraverts, and older people. Second, the two scales of the TIBACh related to belief in divine control during the pandemic in different ways. While belief in divine control during the pandemic predicted a better outcome in terms of positive affect, belief in control during the pandemic was unrelated to change in negative affect.

The fourth conclusion addresses the primary research question posed by this study, namely the connection between self-perceived change in psychological well-being during the pandemic and belief in divine control over the pandemic. Employing the balanced affect model of change in psychological well-being has demonstrated that belief in divine control over the pandemic serves to enhance positive change in positive affect but does nothing to mitigate negative change in negative affect. This finding is worth closer scrutiny alongside two other research findings. First, data from this study has demonstrated that conservative doctrine and charismatic influence function in the same way as belief in divine control. Both conservative doctrine and charismatic influence serve to enhance positive change in positive affect, but do nothing to mitigate negative change in negative affect.

Second, these findings are consistent with the way in which conservative Christian beliefs function in relation to the two components of the Francis Burnout Inventory, as presented by Francis, Haley, and McKenna (2022). Drawing on data provided by 803 Methodist circuit ministers serving in Britain, they tested the association between conservative Christian beliefs and the two scales proposed by the Francis Burnout Inventory as described by Francis et al. (2011): the Emotional Exhaustion in Ministry Scale (negative affect) and the Satisfaction in Ministry Scale (positive affect). After taking into account the effects of personal factors (sex and age), psychological factors (extraversion and neuroticism), contextual factors (married and number of churches), and experience factors (years in ministry and years in current post), holding conservative Christian belief was associated with a higher level of positive affect (satisfaction in ministry) but independent of negative affect (emotional exhaustion in ministry).

Both conservative Christian belief and belief in divine control during the pandemic convey a sense of confidence in the Christian revelation and the assurance of divine omnipotency. The issue of psychological interest is why this style of belief should enhance positive affect but not at the same time mitigate negative affect. The following theory offers an account of this phenomenon as operationalised within the model of psychological well-being proposed by the Francis Burnout Inventory. The negative affect of emotional exhaustion in ministry is an experience over which clergy may have relatively little control. The relentless nature of the emotional demands of their vocation may be less amenable to modification by the narrative of divine omnipotency. The positive affect of satisfaction in ministry, on the other hand, may be more readily nuanced by the narrative of divine omnipotency. Clergy who conceptualise their vocation as collaborators alongside the God who is in control may both pattern and interpret the daily experiences of ministry in a more positive light. It appeared that theological beliefs really do matter in relation to how well clergy survive the emotional stresses of their vocation, an idea that would benefit from further research. The balanced affect model of psychological well-being maintains that positive affect is able to mitigate the debilitating consequence of negative affect (see Village et al., 2018). While belief in divine control during the pandemic may have done nothing to lessen increases in negative affect, the debilitating consequences of the increase in negative affect may have been mitigated by the way in which belief in divine control was reflected in growth in positive affect. According to the present data such mitigation was effective for lay people and for clergy in pursuit of their respective Christian vocations.

### Limitations of the study

This study assessed the impact of belief in divine control during the pandemic on changes in psychological well-being, and it did so by self-reported measures of change in positive affect and change in negative affect, relying on cross-sectional rather than longitudinal measures of psychological well-being. This design is not as robust as would have been the case were we to have been able to measure psychological well-being among the same participants before and after the onset of the pandemic. Self-report measures of this nature are useful for modeling effects on cross-sectional data and provide the only viable option within the current pandemic. Future studies in times of crisis in the Church of England would be helped by having ongoing long-term panel studies conducted among both clergy and lay people.

### Future research

This study suggests that the beliefs associated with the theological notion of divine providence may still be important to many Christians today. The pandemic highlighted the importance of such beliefs in a time of national emergency, but there has been little empirical study of how they operate under “normal” conditions. Future research could explore this area of belief by developing empirical measures that define the contours of belief in divine intervention among clergy and churchgoers. Such scales could be used to inform the construction of versions of the GiCoPS suitable for particular crisis events. They would also enable better understanding of how beliefs in providence are distributed in different populations, and how far specific interventionist beliefs are associated with better psychological or spiritual well-being.
